# MicroRNA-mediated regulation of autophagy in lung cancer: role in tumourigenesis and chemotherapy resistance

**DOI:** 10.20935/acadonco7887

**Published:** 2025-09-11

**Authors:** Yin Yin Sheng, Abdul L. Shakerdi, Graham P. Pidgeon

**Affiliations:** 1 School of Medicine, Trinity Translational Medicine Institute, St. James’s Hospital and Trinity College Dublin, Dublin, Ireland.; 2 Department of Surgery, Trinity Cancer Institute, St. James’s Hospital and Trinity College Dublin, Dublin, Ireland.

**Keywords:** lung cancer, autophagy, microRNAs, therapy resistance, chemoresistance, biomarkers

## Abstract

Autophagy is an evolutionarily conserved catabolic process that enables cells to degrade and recycle long-lived proteins and damaged organelles, playing an important role in cellular homeostasis and survival under stress. In lung cancer, autophagy has emerged as a key modulator of tumour cell survival and is a significant factor influencing chemotherapy efficacy. However, the contribution of autophagy to chemoresistance remains complex and incompletely understood. Recent studies identify microRNA (miRNAs), a class of small non-coding RNAs, as critical regulators of autophagy, capable of modulating autophagy-related genes and signalling pathways. Through this regulatory capacity, miRNAs can alter autophagic activity in lung cancer cells, thereby influencing both chemosensitivity and the development of chemoresistance. This review summarises current advances in the understanding of the miRNA-mediated regulation of autophagy in the context of lung cancer, with particular emphasis on its impact on chemotherapy response. Mechanistic insights into how miRNAs govern specific stages of the autophagic process are examined, and the potential for therapeutic intervention targeting the miRNA–autophagy axis to mitigate chemoresistance in lung cancer is discussed.

## 1. Introduction

Lung cancer is the leading cause of cancer-related mortality worldwide, accounting for an estimated 2.2 million new cases and 1.8 million deaths annually [1]. Despite significant advances in targeted therapies and immunotherapy, the overall five-year survival rate for lung cancer remains approximately 20% [1]. This poor prognosis is predominantly attributable to late-stage diagnosis and therapeutic resistance [2, 3]. Autophagy is a highly conserved catabolic process through which cells degrade and recycle cytoplasmic components, including long-lived proteins that are susceptible to damage and aggregation, which can impair proteostasis [4]. Their clearance prevents proteotoxic stress, while the recycling process provides essential metabolites and removes dysfunctional organelles, thereby supporting homeostasis and survival under stress [5]. In the context of cancer, autophagy plays a paradoxical and context-dependent role. In the early stages of tumourigenesis, autophagy functions as a tumour suppressor by eliminating damaged organelles and misfolded proteins, preserving genomic stability [6]. Conversely, in established tumours, autophagy is co-opted by cancer cells to support survival under conditions of hypoxia, nutrient deprivation, and therapeutic stress [6]. Recent studies have highlighted the role of microRNAs (miRNAs) as key post-transcriptional regulators of autophagy in lung cancer [7, 8]. These small non-coding RNAs modulate the expression of autophagy-related genes and signalling pathways, thereby influencing tumour progression, therapeutic response, and chemoresistance. While the role of autophagy in cancer biology is well established, to our knowledge, the specific mechanisms through which miRNAs regulate autophagy to drive drug resistance in lung cancer have not been systematically addressed. This review therefore examines the emerging evidence on miRNA–autophagy interactions in lung cancer and their implications for overcoming therapy resistance, with a view of identifying potential biomarkers and novel therapeutic strategies.

## 2. Molecular mechanisms of autophagy

Autophagy encompasses three mechanistically distinct subtypes, namely macroautophagy, microautophagy, and chaperone-mediated autophagy (CMA) [9]. Among these, macroautophagy is the best characterised, involving the sequestration of cytoplasmic material within double-membraned autophagosomes that subsequently fuse with lysosomes for cargo degradation and recycling [9]. In contrast, microautophagy involves the direct invagination of the lysosomal membrane without the intermediate formation of autophagosomes [10]. CMA represents a highly selective form of autophagy, whereby it targets soluble proteins bearing a KFERQ-like motif for translocation across the lysosomal membrane via the lysosome-associated membrane glycoprotein 2A (LAMP-2A) receptor, following recognition by heat shock cognate protein 70 (Hsc70) [11].

**Figure 1 •:**
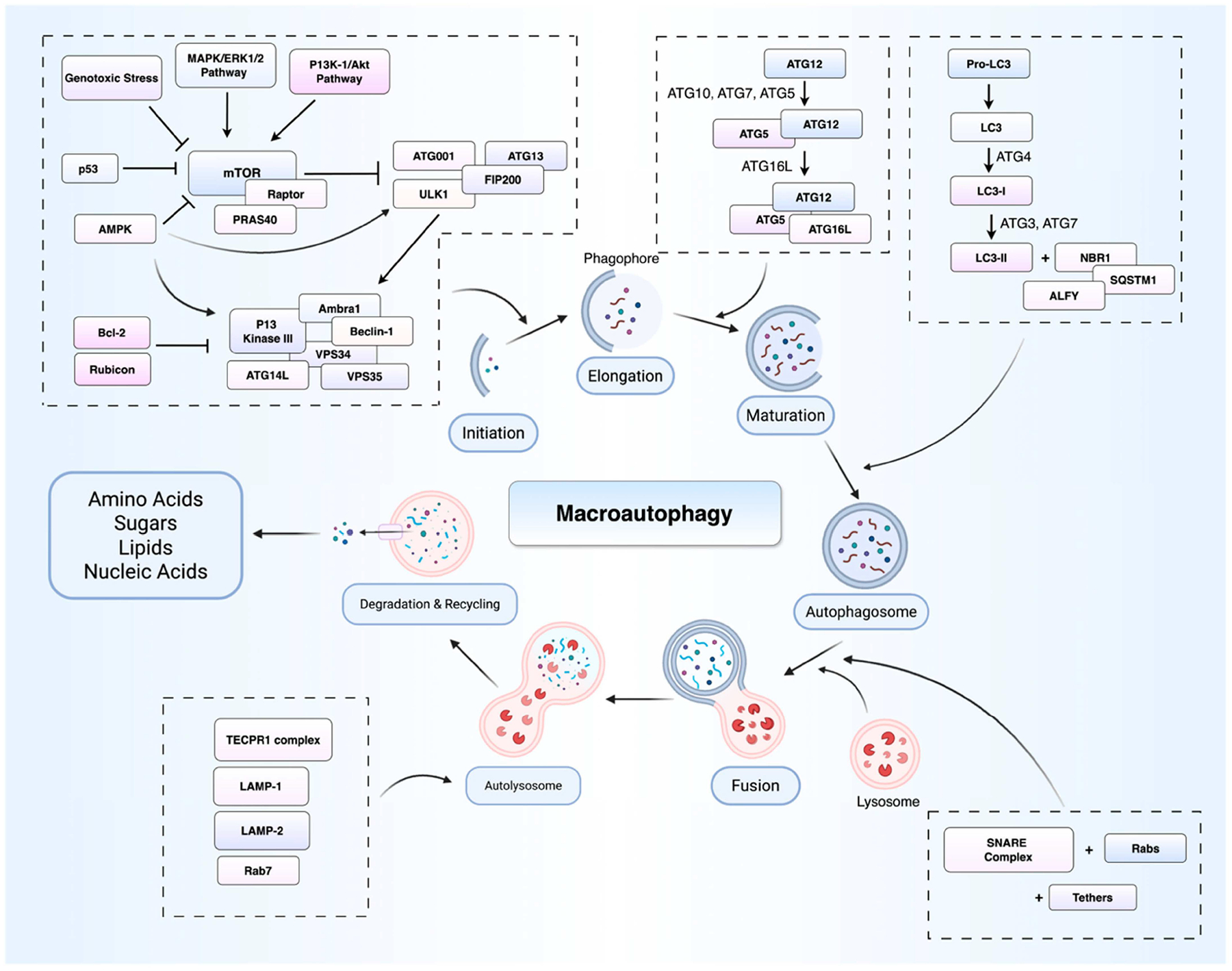
Overview of the macroautophagy pathway. This schematic illustrates the sequential steps of macroautophagy. Upon exposure to cellular stress like nutrient deprivation or hypoxia, the ULK1/2 initiation complex is activated, triggering the formation of the phagophore. The phagophore membrane expands with the involvement of autophagy-related proteins (ATGs) and the Beclin1-Vps34 complex, which generate phosphatidylinositol 3-phosphate and recruit additional factors. The ATG5-ATG12-ATG16L conjugation system and LC3 processing facilitate phagophore elongation and cargo selection, leading to the formation of a double-membraned autophagosome that encloses targeted cytoplasmic material. A mature autophagosome subsequently fuses with a lysosome to form an autolysosome, where lysosomal enzymes degrade the sequestered contents for recycling and cellular homeostasis maintenance. This pathway is fundamental to cell survival, and its dysregulation is implicated in lung cancer. ATG—autophagy-related gene; Bcl—B-cell lymphoma; MAPK—mitogen-activated protein kinase; ERK—extracellular signal-regulated kinase; P13K—phosphoinositide 3-kinase; ULK—Unc-51 like autophagy activating kinase 1; AMPK—AMP-activated protein kinase; VPS—vacuolar protein sorting; LAMP—lysosome-associated membrane protein; RAB—Ras-related proteins; LC3—microtubule associated protein 1 light chain 3; SNARE—soluble N-ethylmaleimide-sensitive factor attachment protein receptor. Created in BioRender. Sheng, Y. (2025). https://BioRender.com/bn5fncg.

These sequential steps of autophagy are orchestrated by more than 40 autophagy-related (ATG) proteins, of which the ATG8/LC3 family plays a central role in autophagosome formation and cargo recruitment [12]. Autophagy induction is primarily regulated by the interplay between mechanistic target of rapamycin complex 1 (mTORC1), a key negative regulator, and AMP-activated protein kinase (AMPK), which activates autophagy by phosphorylating Unc-51-like autophagy activating kinase 1 (ULK1) [13]. The ULK1 complex initiates autophagosome formation, while the class III P13K complex (Beclin 1, Vps34, Vps15, and ATG14) mediates membrane nucleation [14]. Selective cargo degradation is achieved through adaptor proteins like p62/SQSTM1, which link ubiquitinated substrates to LC3 on autophagosomes [15] (Figure 1).

Membrane elongation and closure are driven by the ATG12-ATG5 and LC3 conjugation systems, with LC3 lipidation (LC3-II) marking autophagosomal membranes for cargo sequestration [9]. Fusion of mature autophagosomes with lysosomes, regulated by soluble N-ethylmaleimide-sensitive factor attachment protein receptor (SNARE) proteins and Rab7, leads to the degradation and recycling of cellular components [4].

## 3. Role of autophagy in lung cancer

Autophagy is frequently characterised as a double-edged sword across multiple cancer types. Various lines of evidence also specifically highlight this dichotomy in lung cancer in cell lines and animal models. Mathew et al. established a mechanistic connection between autophagy, cellular metabolism, and genomic stability using Beclin 1- and Atg5-deficient cell models [16]. Employing assays like PALA resistance, polymerase chain reaction (PCR), and array comparative genomic hybridization (aCGH), they demonstrated that autophagy-deficient cells exhibit reduced survival under metabolic stress conditions [16]. Paradoxically, this survival disadvantage is counterbalanced by an increase in mutation rates and chromosomal instability, factors that can potentiate tumourigenesis [17]. For example, serum deprivation, a known metabolic stressor, induces autophagy, while defects in this response in zebrafish embryonic fibroblasts, a non-cancer model, lead to heightened DNA damage and gene amplification [17]. These findings underscore the essential role of both constitutive and stress-induced autophagy in preserving genomic integrity and limiting oncogenic transformation.

Complementary in vivo evidence from murine models of lung cancer with the deletion of Atg5 in pneumocytes revealed that autophagy serves a dual role in lung carcinogenesis [18]. The early loss of Atg5-dependent autophagy significantly accelerated tumour initiation, resulting in an increased number of tumour foci and a more rapid progression from hyperplasia to benign adenomas [18]. However, loss of Atg5-dependent autophagy impaired malignant progression, as tumours showed reduced proliferation, increased apoptosis and necrosis, impaired mitochondrial function, and elevated DNA damage responses [3].

The shift to an oncogenic role of autophagy in established tumours is particularly evident in RAS-driven lung cancer, which frequently harbours activating mutations in the *KRAS* oncogene [19]. These tumours display a phenomenon termed “autophagy addiction”. characterised by constitutively elevated autophagic flux that supports sustained proliferation under nutrient-limiting conditions [19]. Recent studies also identify non-canonical autophagy pathways in RAS-driven tumours, such as PI4KB/ULK1-dependent autophagosome formation, which operate independently of classical ATG proteins [19]. The pharmacological inhibition of autophagy has emerged as a potential therapeutic approach to target this vulnerability. For example, hydroxychloroquine (HCQ), a lysosomotropic agent that impairs autophagosome degradation by disrupting lysosomal acidification, has been shown to sensitise RAS-mutant lung cancer cells to chemotherapy and targeted therapies [20, 21]. Products of autophagic degradation such as amino acids, fatty acids, and other metabolites can serve as substrates for mitochondrial metabolic pathways such as the tricarboxylic acid (TCA) cycle, particularly under nutrient-deprivation conditions [19]. Thus, the inhibition of autophagy could limit the availability of these substrates and attenuate the growth of RAS-mutant lung cancer cells [19]. In a parallel mechanism of resistance, Hu et al. demonstrated that lncRNA MITA1 is significantly upregulated in gefitinib-resistant HCC827GR lung cancer cells [22]. Gefitinib is a targeted cancer drug, specifically an oral, small molecular tyrosine kinase inhibitor of the epidermal growth factor receptor, primarily used in the treatment of NSCLC with activating EGFR mutations [22]. Increased MITA1 expression promotes gefitinib resistance in NSCLC by enhancing autophagy, which suppresses apoptosis and supports cell survival under treatment stress [22, 23]. Conversely, siRNA-mediated LAMP-2A depletion can inhibit autophagy, reduce lung cancer cell proliferation, and enhance cisplatin sensitivity [24]. However, many studies utilise LAMP-2A expression as a surrogate for CMA activity without direct functional validation, and targeting LAMP-2A is complicated by its similarity to other LAMP2 isoforms with distinct roles. Thus, the choice of autophagy inhibition strategy may require careful consideration of context and tumour stage.

An emerging area of interest lies within the correlation between hypoxia and autophagy in lung cancer. Under hypoxic conditions, autophagy-related proteins such as LC3-II and BNIP3 are upregulated, increasing autophagic flux and reducing cisplatin-induced apoptosis [25]. The inhibition of autophagy with agents like 3-MA can restore chemosensitivity under hypoxia, highlighting its protective role [26]. While BNIP3 overexpression can trigger cell death, its regulation is complex and context-dependent, with autophagy modulating BNIP3 and BNIP3L levels and influencing treatment response [26]. Excessive autophagy induction through bupivacaine and guaiazulene under hypoxia can also lead to autophagic cell death through the suppression of lung cancer cell proliferation [27]. Autophagy conventionally serves as a cytoprotective mechanism, facilitating cellular survival under metabolic stress by recycling intracellular components [28]. However, when autophagic activity is sustained or dysregulated, it can lead to the degradation of crucial organelles and proteins, thereby shifting cellular balance towards cell death. The molecular determinants that dictate whether autophagy promotes cell survival or induces cell death remain incompletely elucidated. These outcomes are modulated by several factors like the magnitude and duration of autophagic flux, the specific cellular context and microenvironment, and key signalling pathways like Akt/mTOR axis [27]. Given its complexity in lung cancer, further research is essential to delineate the precise mechanisms by which these variables influence autophagic outcomes.

## 4. miRNA in the pathogenesis of lung cancer

miRNAs are small, non-coding RNA molecules typically 20–24 nucleotides long that regulate gene expression post-transcriptionally by binding to complementary sequences on target messenger RNAs (mRNAs), leading to their degradation or translational repression [29]. They regulate the expression of various genes involved in processes like cell differentiation, proliferation, and apoptosis [30]. For instance, miR-15 and miR-16 induce apoptosis by targeting the major anti-apoptotic gene BCL2 [31]. The dysregulation of miRNA is a hallmark of many cancers, including lung cancer [30]. For instance, miR-21 is consistently overexpressed in NSCLC, promoting tumourigenesis by targeting tumour suppressors like PTEN and PDCD_4_ [32]. Its expression correlates with poor outcomes, while anti-miR-21 treatment induces apoptosis and S-phase arrest in the A549 lung cancer cell line [33, 34]. Conversely, tumour-suppressive miRNAs like miR-34a and miR-200c are frequently downregulated [35]. miR-34a exerts its anti-tumour effects by targeting the MET proto-oncogene and BCL2, key regulators of cancer progression [35]. MET encodes a receptor tyrosine kinase that mediates signalling pathways involved in cell proliferation, survival, migration, and invasion and is frequently dysregulated in lung cancer [35]. By suppressing MET, miR-34a inhibits oncogenic signalling cascades that drive tumour growth and metastasis [35]. Concurrently, miR-34a targets BCL2, thereby promoting apoptosis and reducing tumour cell survival [35]. In parallel, miR-200c plays a critical role in suppressing the epithelial-to-mesenchymal transition (EMT) [36]. This underscores the critical role of miRNA dysregulation in the pathogenesis of lung cancer and highlights its potential as both biomarkers and therapeutic targets.

## 5. miRNA as regulators of chemosensitivity in lung cancer

### 5.1. miR-1

miR-1, a recognised tumour-suppressive microRNA, regulates the key hallmarks of cancer, including cell proliferation, survival, apoptosis, and chemosensitivity [37]. It is consistently downregulated in tumour tissues, serum samples, and cell lines of small-cell lung cancer (SCLC) [38]. Khan et al. (2023) demonstrated that the CXCR4/FOXM1/RRM2 axis drives SCLC growth and metastasis via transcriptional activation of the RRM2 promotor [38]. Hua and colleague identified miR-1 as a negative regulator of cisplatin resistance in NSCLC [7]. Functional analyses demonstrate that miR-1 overexpression suppresses autophagy, as indicated by reduced LC3B-II/LC3B-I ratios, elevated p62 levels, and decreased GFP-LC3 puncta formation [7, 38]. miR-1 directly targets ATG3, which is upregulated in resistant cells, leading to the inhibition of ATG3-mediated autophagy [7]. The restoration of ATG3 expression reverses the effects of miR-1, reinstating autophagic flux and diminishing apoptosis, as well as reducing cisplatin sensitivity [7]. Collectively, these findings suggest that miR-1 enhances cisplatin responsiveness in NSCLC by suppressing ATG3-driven autophagy, highlighting the miR-1/ATG3 axis as a promising therapeutic target for overcoming chemoresistance.

### 5.2. miR-15a

miR-15a, located on chromosome 13q14, was among the first miRNAs identified as being associated with cancer and is notably downregulated in lung cancer tissues and cell lines [39]. Both miR-15a-3p and miR-15a-5p exhibit tumour-suppressive properties by inhibiting the proliferation, migration, and invasion of lung cancer cells [40]. The overexpression of miR-15a in NSCLC cells significantly reduces cell viability, migration, and invasion while promoting apoptosis with no apparent effect on cell cycle progression [39]. miR-15a directly targets and negatively regulates anti-apoptotic genes like BCL2L2 and BCL2, as well as Smad3, thereby suppressing tumour progression and metastasis [40]. miR-15a-5p also suppresses lipid metabolism and metastasis by targeting and downregulating ACSS2, thereby reducing fatty acid synthesis and acetyl-CoA activity [41]. Importantly, the loss of miR-15a-3p is linked to the development of cisplatin resistance in lung cancer, while restoration of its expression sensitises resistant cells to cisplatin by promoting apoptosis and autophagy, potentially through BCL2 suppression [42].

### 5.3. miR-16, miR-17

Both miR-16 and miR-17 were found to be associated with chemoresistance in lung cancer through its downregulation in paclitaxel-resistant lung cancer cells [43]. This dual downregulation appears to drive resistance through the inhibition of apoptosis and activation of cytoprotective autophagy [43]. miR-16 was shown to directly target the anti-apoptotic protein Bcl-2, and its downregulation led to Bcl-2 upregulation, inhibiting apoptosis and promoting resistance [43, 44]. Similarly, the downregulation of miR-17 resulted in an increased expression of Beclin-1, enhancing autophagy and further contributing to resistance [43]. Studies also implicate lncRNA BLACAT1 in promoting cisplatin resistance in NSCLC through the modulation of autophagy pathways [44]. BLACAT1 is significantly upregulated in cisplatin-resistant NSCLC cells and acts as a molecular sponge for miR-17, thereby relieving repression of the autophagy-related gene ATG7 [44]. This repression leads to increased expression of autophagy markers, including LC3-II and Beclin-1, and enhances autophagic activity, which contributes to chemoresistance [45]. The simultaneous downregulation of both miRNAs exacerbated its effect and facilitated paclitaxel resistance by promoting autophagy and inhibiting apoptosis [45]. Co-overexpression of both miRNAs resulted in a synergistic effect, significantly promoting apoptosis and lowering the effective paclitaxel dose required [45]. Mechanistically, this was accompanied by suppression of the PI3K/Akt/mTOR survival pathway, decreased mitochondrial membrane potential, and a substantial increase in reactive oxygen species.

### 5.4. miR-30

miR-30 has emerged as a notable tumour suppressor in lung cancer, exerting its effects by inhibiting cell proliferation, migration, and autophagy. In SCLC, Beclin-1 is commonly upregulated in drug-resistant cells, contributing to enhanced autophagic flux and chemoresistance [46]. Yang et al. demonstrated that transfection with miR-30a-5p suppresses Beclin-1 expression, effectively inhibiting autophagy and restoring sensitivity in SCLC cells to etoposide and cisplatin [46]. Beyond Beclin-1, miR-30a-5p targets additional oncogenic and autophagy-related genes including SIRT1, SOX4, and components of the P13K/AKT pathway, thereby influencing cell cycle progression, apoptosis, and EMT [47]. These findings highlight the multifaceted role of miR-30a-5p in modulating both autophagy and chemoresistance. However, given the complexity of autophagy, further investigation is warranted to elucidate additional downstream targets and pathways through which miR-30a-5p may influence therapeutic response.

### 5.5. miR-223

**Figure 2 •:**
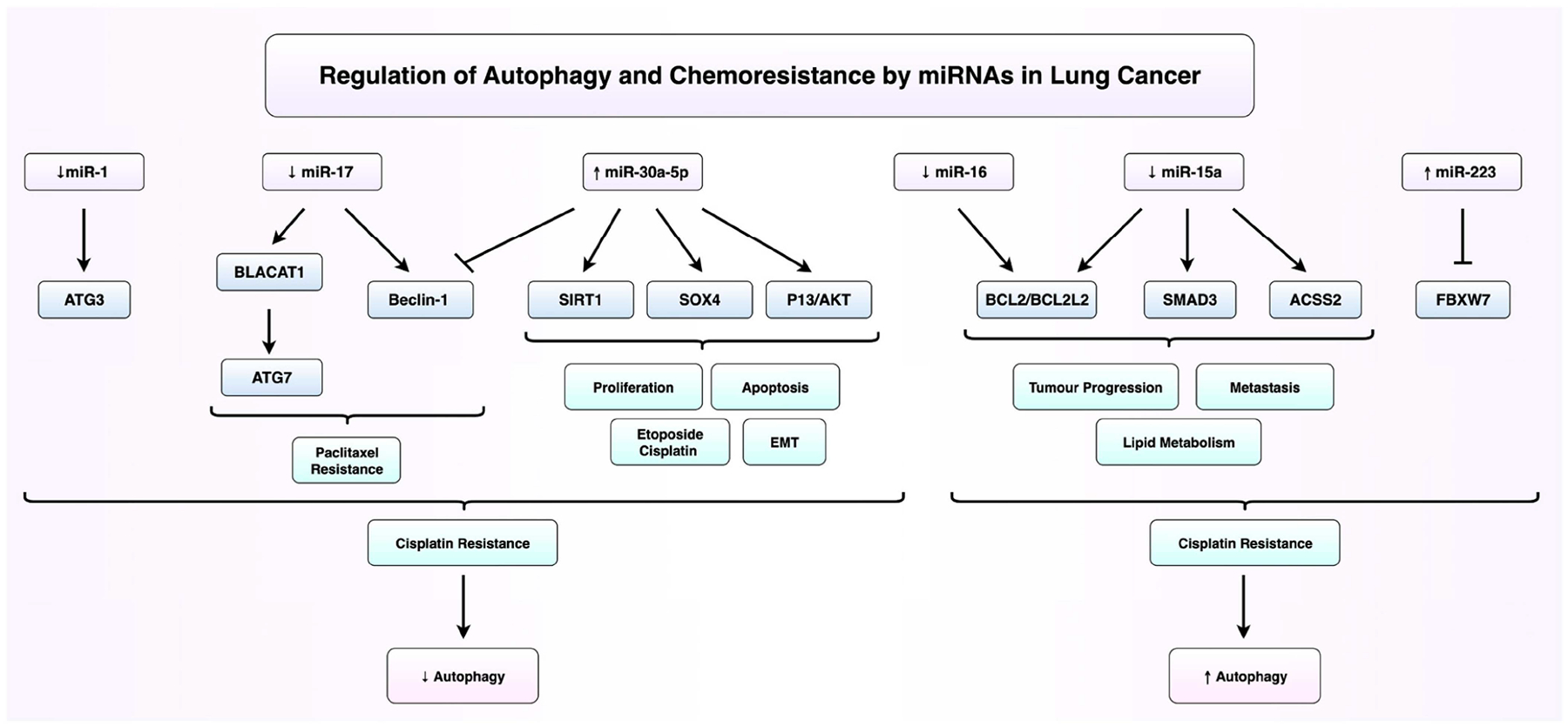
miRNA regulation of drug resistance and autophagy in lung cancer. This schematic illustrates key microRNAs (miRNAs) involved in modulating autophagy and chemoresistance in lung cancer. Upregulated (↑) and downregulated (↓) miRNAs are indicated to reflect their expression changes in lung cancer. Tumour-suppressive and oncogenic miRNAs shown in the diagram either activate or inhibit autophagy-related genes, thereby promoting or inhibiting apoptosis. This also induces resistance to chemotherapy including etoposide, paclitaxel, and cisplatin. This diagram portrays the complex connection between the miRNA-mediated regulation of autophagy and its impact on lung cancer therapeutics, highlighting potential targets for overcoming chemoresistance. miR—microRNA; P13K—phosphoionositide 3-kinase; AKT—Ak strain transforming; BLACAT—bladder cancer-associated transcript; ATG—autophagy-related genes; BCL—B-cell leukaemia/lymphoma 2; ACSS2—acetyl-CoA synthetase 2; FBXW7—F-box and WD repeat domain containing 7. Created in BioRender. Sheng, Y. (2025). https://BioRender.com/q1oly94.

Recent studies have identified miR-223 as a significant mediator of inflammation and cisplatin resistance in NSCLC [48]. While miR-223 was previously linked to doxorubicin resistance, current evidence demonstrates that its overexpression in NSCLC cells also reduces sensitivity to cisplatin [49]. Mechanistically, miR-223 directly targets the tumour suppressor FBXW7, leading to its downregulation and a subsequent increase in autophagic activity within NSCLC [49]. Functional assays confirm that elevated miR-223 enhances cell survival under cisplatin treatment, whereas the inhibition of miR-223 restores cisplatin sensitivity both in vitro and in xenograft models [49]. These findings highlight miR-223 as a promising therapeutic target for overcoming cisplatin resistance in NSCLC by modulating autophagy pathways. Figure 2 provides a summary of the key mechanisms by which miRNAs regulate chemoresistance in lung cancer through the modulation of autophagy.

## 6. Biomarker potential, therapeutic implications, and future direction

Autophagy-related signatures hold promise as prognostic biomarkers in lung cancer. A study by Jiang et al. (2021) identified 1144 autophagy-related lncRNAs using transcriptomic and clinical data from The Cancer Genome Atlas (TCGA) and autophagy-related genes from the Human Autophagy Database and developed a 16-lncRNA prognostic model that stratified LUAD patients into high- and low-risk groups, with high-risk patients showing poorer survival [50]. TMPO-AS1 and BIRC5 emerged as key components, correlating with advanced disease stage and poor prognosis. Similarly, lncRNA LUCAT1 is upregulated in cisplatin-resistant NSCLC, where it binds miR-514a-3p, relieving the inhibition of ULK1 to enhance autophagy [51]. As such, further studies should clarify the mechanistic roles of such to refine their prognostic and therapeutic value. Prognostic studies combine gene expression profiles with clinical data to identify molecular signatures that predict patient survival or disease progression, often using multivariate Cox regression to stratify patients by risk [52]. Given autophagy’s dual role in cancer, these models capture its complex influence. Elucidating how lncRNAs modulate autophagy will help refine their prognostic and therapeutic value in lung cancer.

Beyond chemotherapy, autophagy influences responses to radiation and targeted therapies in lung cancer. For example, lncRNA KCNQ1OT1 promotes radioresistance in LUAD via ATG5/ATG12-mediated autophagy, while HOTAIR overexpression induces resistance to the ALK inhibitor crizotinib by activating ULK1 [53]. Silencing HOTAIR inactivated autophagy through the suppression of ULK1 phosphorylation and decreased drug resistance. Supporting this, the induction of autophagy using rapamycin abolished the drug-sensitising effect of HOTAIR silencing [53]. Although direct evidence for miRNA-mediated autophagy in lung cancer radioresistance is limited, its role is plausible and requires further investigation.

MicroRNA function can be therapeutically modulated in cancer either by miRNA mimics, which restore or enhance the function of downregulated tumour-suppressive miRNAs, or by miRNA inhibitors, which block oncogenic miRNAs [54]. Preclinical lung cancer models have demonstrated that miRNA mimics or inhibitors can suppress tumour growth and reverse drug resistance. For example, the systemic delivery of miR-200c, which negatively regulates the EMT master regulator ZEB1, enhances radiosensitivity in a lung cancer xenograft model [55]. In addition, restoring miR-16 in NSCLC cells not only inhibits key oncogenic pathways such as ERK/MAPK but also renders the cancer cells more susceptible to cisplatin and EGFR inhibitors in xenograft models [8]. Nevertheless, translating miRNA therapeutics into clinical applications has proven challenging due to various limitations, and thus far, no miRNA-based therapy has achieved clinical approval in lung cancer. As miRNAs often regulate a wide range of mRNAs, therapeutic targeting can come with significant off-target events. Safety concerns were raised during the first in-human phase I clinical trial of the miR-34a mimic MRX34 in advanced solid tumours, which was terminated early following significant serious adverse events including cytokine release syndrome and four patient deaths [56].

Therapeutic delivery of miRNAs remains challenging, as naked miRNAs are rapidly degraded in the bloodstream and may not efficiently reach the tumour [57]. Various chemical modifications and delivery systems are being investigated to overcome this, including liposomal formulations and ligand-targeted nanoparticles, but each adds additional complexity and potential toxicity [57]. Lipid-based nanoparticles may trigger immune responses or accumulate in non-target tissues [57]. Furthermore, the abnormal vasculature and microenvironment of tumours could impede effective delivery and distribution of therapeutic miRNAs. Beyond delivery, the pleiotropic nature of miRNA can pose further challenges as a single miRNA can regulate multiple genes and pathways, which increases the risk of unintended effects [58].

While it remains unclear whether systemic miRNA delivery can selectively target malignant cells without affecting normal tissues, new delivery platforms exploit tumour-specific markers and microenvironmental factors to enhance specificity. Nanoparticles with ligands for cancer cell receptors or tumour-enriched miRNA profiles help limit off-target effects [59, 60]. Additionally, “nor-momiRs”, miRNAs abundant in normal tissues but downregulated in tumours, may offer safer therapeutic windows [58]. Despite these promising strategies, comprehensive preclinical and clinical evaluations are needed to fully assess the safety and efficacy of miRNA-based therapies.

## 7. Conclusions

This review highlights how the intricate interplay between microRNAs and autophagy plays a pivotal role in modulating tumourigenesis and chemotherapy response in lung cancer. While autophagy dysregulation has been implicated across a wide range of cancers, the specific mechanisms by which miRNAs regulate autophagy-driven chemoresistance in lung cancer remain comparatively underexplored. This relationship may offer promising targets and biomarkers for therapeutic interventions and patient stratification in clinical settings. Despite promising preclinical findings, the clinical translation of miRNA-based therapies remains challenging due to issues primarily related to delivery and safety. Future research should prioritise the development of robust delivery systems, identification of predictive biomarker panels, and integration of miRNA–autophagy signatures into personalised treatment strategies. Continued innovation in these areas holds significant promise for advancing miRNA-based interventions and improving outcomes for lung cancer patients.

## Data Availability

All data supporting the findings of this publication are available within this article.
